# Effectiveness and safety of basal insulin therapy in type 2 diabetes mellitus patients with or without metformin observed in a national cohort in China

**DOI:** 10.1186/s12902-021-00892-6

**Published:** 2022-01-19

**Authors:** Puhong Zhang, Minyuan Chen, Heng Zhang, Yingying Luo, Dongshan Zhu, Xian Li, Jiachao Ji, Du Wang, Nadila Duolikun, Linong Ji

**Affiliations:** 1 The George Institute for Global Health at Peking University Health Science Center, 100600 Beijing, China; 2 Faculty of Medicine, University of New South Wales, Sydney, Australia; 3 Department of Endocrinology and Metabolism, Peking University People’s Hospital, 100040 Beijing, China

## Abstract

**Background:**

Though many randomized control trials had examined the effectiveness and safety of taking insulin therapy with or without metformin, there are limited real-world data, especially among Chinese type 2 diabetes patients initiating basal insulin (BI) with uncontrolled hyperglycemia by oral agents. This study was designed to assess the effectiveness and safety of BI therapy combined with or without metformin in a real-world national cohort study.

**Methods:**

Patients with type 2 diabetes mellitus who initiated BI treatment due to uncontrolled hyperglycemia (HbA1c≥7 %) by oral antidiabetic drugs (OADs) were recruited in Chinese real-world settings between 2011 and 2013. A total of 12,358 patients initiated BI without bolus insulin and completed a 6-month follow-up were selected as the study population and divided into BI with metformin or BI without metformin group based on whether metformin was simultaneously prescribed or not at baseline. Propensity score adjustment was used to balance baseline covariates between two groups. A sub-analysis was also conducted among 8,086 patients who kept baseline treatment regimen during the follow-up. Outcomes were HbA1c, hypoglycemia, weight gain and insulin dose in two groups.

**Results:**

53.6 % (6,621 out of 12,358) patients initiated BI therapy concomitant with metformin. After propensity score adjustment, multivariate regression analysis controlled with number of OADs, total insulin dose, physical activity and diet consumption showed that BI with metformin group had a slightly higher control rate of HbA1c <7.0 % (39.9 % vs. 36.4 %, *P *= 0.0011) at 6-month follow-up, and lower dose increment from baseline to 6-month (0.0064 vs. 0.0068 U/day/kg, *P *= 0.0035). The sub-analysis with patients remained at same BI therapy further showed that BI with metformin group had higher HbA1c control rate (47.9 % vs. 41.9 %, *P *= 0.0001), less weight gain (-0.12 vs. 0.15 kg *P *= 0.0013), and lower dose increment during 6-month follow-up (0.0033 vs. 0.0037 U/day/kg, *P *= 0.0073) when compared with BI without metformin group.

**Conclusions:**

In alliance with current guidelines, the real-world findings also support the insulin initiation together with metformin. Continuous patients’ education and clinicians training are needed to improve the use of metformin when initiating BI treatment.

## Background

With estimated 113.9 million people in China and 420 million globally affected, diabetes continues to be one of the most common non-communicable diseases and public health priorities worldwide [1, 2]. Type 2 diabetes mellitus (T2DM) is the main category of diabetes in adults, which is characterized by steady deterioration of glycemic control due to progressive β-cell dysfunction of insulin secretion frequently on the background of insulin resistance. The objective of T2DM therapy is to safely achieve and maintain glycemic control in order to reduce the risk of microvascular and macrovascular complications, and in the long run, diabetes related mortality. However, as achieving a near-normal glycemic control target becomes increasingly difficult due to disease progression, most patients ultimately require insulin treatment [3–5].

Although timely addition of insulin to oral therapy for T2DM has been recommended to prevent complications by early institution of strict glycemic control and β-cell protection [3], a substantial proportion of patients with suboptimal glucose control tend to delay insulin therapy due to fear of hypoglycemia, along with a reluctance to start injections and gain weight [6], which may further influence adherence to insulin therapy [7]. Metformin as first line therapy for T2DM is recommended to be continued after initiating insulin (usually basal insulin (BI)) by current guidelines [8–10] since the regimen of insulin plus concomitant metformin was shown to improve glycemic control, reduce hypoglycemia incidence and weight gain as well as total insulin dose when compared with insulin monotherapy [11–14]. In addition, this combination regimen was reported to reduce the risk of death and macrovascular events compared with insulin monotherapy in the long run [15]. Though many randomized control trials had examined the effectiveness and safety of taking insulin therapy with or without metformin [14, 16–18], and current guidelines recommend metformin to be the first initial oral agent and should be continued after insulin initiation except for contraindication [9, 10], real-world studies from U.S. showed that only around 71.7 % [19] and 72.7 % [20] patients were actually taking metformin before insulin initiation, and dropped to approximately 63.8 % [19] and 72.5 % [20] after insulin initiation. To our knowledge, there is no study among Chinese population reporting metformin use and effectiveness before and after insulin initiation.

Therefore, based on the data of the Observational Registry for BI Treatment (ORBIT) study [21], we analyzed the combination of metformin with BI initiation, and compare the effectiveness and safety between BI users with and without metformin among Chinese population in real-world, with the ultimate purpose of improving the practical adherence to guidelines on metformin use and identifying further researches to improve diabetes care during insulin transition.

## Methods

### Study design and participants

ORBIT was a real-world cohort study conducted from 2011 to 2013. Patients in age of 18–80 years and initiating BI treatment due to inadequately HbA1c control (HbA1c ≥ 7 %) by OADs were recruited from 209 hospitals in Mainland China and followed up for 6 months. Details on the study design were reported previously [21]. The ORBIT protocol was approved by the Institutional Review Board (IRB) of Peking University (IRB. No. IRB00001052-11070) and, when necessary, by local IRBs. Written informed consents were obtained from all patients.

### Data collection and data used for this study

Study data were collected through interview at baseline (visit 1), 3 months (visit 2) and end of the study (6-month, visit 3), including demographics (gender, age, education, patient recruitment settings, current residence and medical insurance), clinical characteristics (diabetes duration, OADs treatment duration before BI therapy), standard physical examination (body weight, height and blood pressure), number and types of OADs before and after initiating BI, hypoglycemia, lifestyle involving food intake and physical activity, and laboratory test of fasting plasma glucose (FPG) and HbA1c at both visit 1 (v1) and visit 3 (v3). Only patients who initiated BI alone (without prandial insulin) and completed the 6-month follow-up were included in this study.

### Grouping method and outcomes

The study population were divided into BI with metformin or BI without metformin group based on whether BI was simultaneously combined with metformin or not at baseline. A sub-analysis was also conducted among patients who remained at baseline treatment of BI with or without metformin throughout the 6 months follow-up. The change of BI therapy was defined as: (1) changing to basal bolus therapy, (2) changing to premix insulin, (3) changing to pure bolus, and (4) discontinuity of insulin therapy. All other BI therapies were regarded as remaining BI treatment regardless of the changes in BI type (changing among Glargine, Detemir and NPH), BI dose and the type of concomitantly used OADs, and also regardless of the variation in the number and timing of insulin injection.

The primary outcomes of the study were control rate of HbA1c (<7.0 % or ≤6.5 %) and incidence of hypoglycemia. Hypoglycemia was categorized as severe hypoglycemia, documented symptomatic, asymptomatic, probable symptomatic or pseudo-hypoglycemia according to the American Diabetes Association recommendations [22]. Self-reported frequency of severe hypoglycemia in the previous 3 months and other minor hypoglycemia in the previous 1 month were documented at each visit. In this study, we reported the incidence of hypoglycemia in terms of incidence density and incidence rate of any kinds of hypoglycemia. The incidence density was calculated as the number of hypoglycemia episodes per person-year, while the incidence rate was defended as the percentage of participants reported at least one of any kind of hypoglycemia at baseline. Secondary outcomes were mean levels of HbA1c, FPG, weight and insulin dose; control rate of FPG (<7.0 mmol/L) at v3; and mean changes of HbA1c, FPG, weight and insulin dose at v3 compared with v1.

### Statistical analyses

Multivariate analysis based on propensity score adjustment was used to compare the outcomes between two groups. Descriptive statistics, including means ± SD for continuous variables and n (%) for categorical variables, were used to present the demographic and clinical characteristics of patients before and after propensity score adjustment respectively. Levels in HbA1c, FPG, body weight and insulin dose were analyzed by using propensity score-adjusted multivariate linear regression models. Rates of hypoglycemia, control rates of HbA1c and FPG were analyzed by using propensity score-adjusted multivariate logistic regression models.

A propensity score was calculated by using logistic regression analysis in this nonrandomized sample; it defines the conditional probability for an individual to be exposed to BI with metformin, on the basis of baseline covariates. The propensity scores were used to adjust for differences of baseline covariates between BI with metformin group and BI without metformin group, including regions, current residence, hospital level, medical insurance (actual proportion of out of pocket), gender, age, body mass index (BMI), duration of diabetes and OAD treatment, HbA1c and FPG levels, hypoglycemia events at baseline, diabetes complication, number and type of OADs prior to BI therapy, and BI types used at baseline [23, 24]. Before and after propensity score adjustment, Pearson’s chi square test and unpaired t-test were used to analyze differences in proportions and continuous variables of baseline values, respectively; balance in variables was indicated when *P* values >0.05 after propensity score adjustment. Process confounding factors which could change during the follow-up were also analyzed, including number of OADs, total insulin dose, physical activity and diet consumption at visit 3. In regression analyses of outcomes, baseline covariates were adjusted by propensity scores and process confounding factors were controlled in regression models.

All reported *P* values were two sided, with *P* values<0.05 considered to be statistically significant. Statistical analyses were performed with Statistical Analysis System, version 9.4 (SAS Institute, Cary, NC, USA).

## Results

**Fig. 1:**
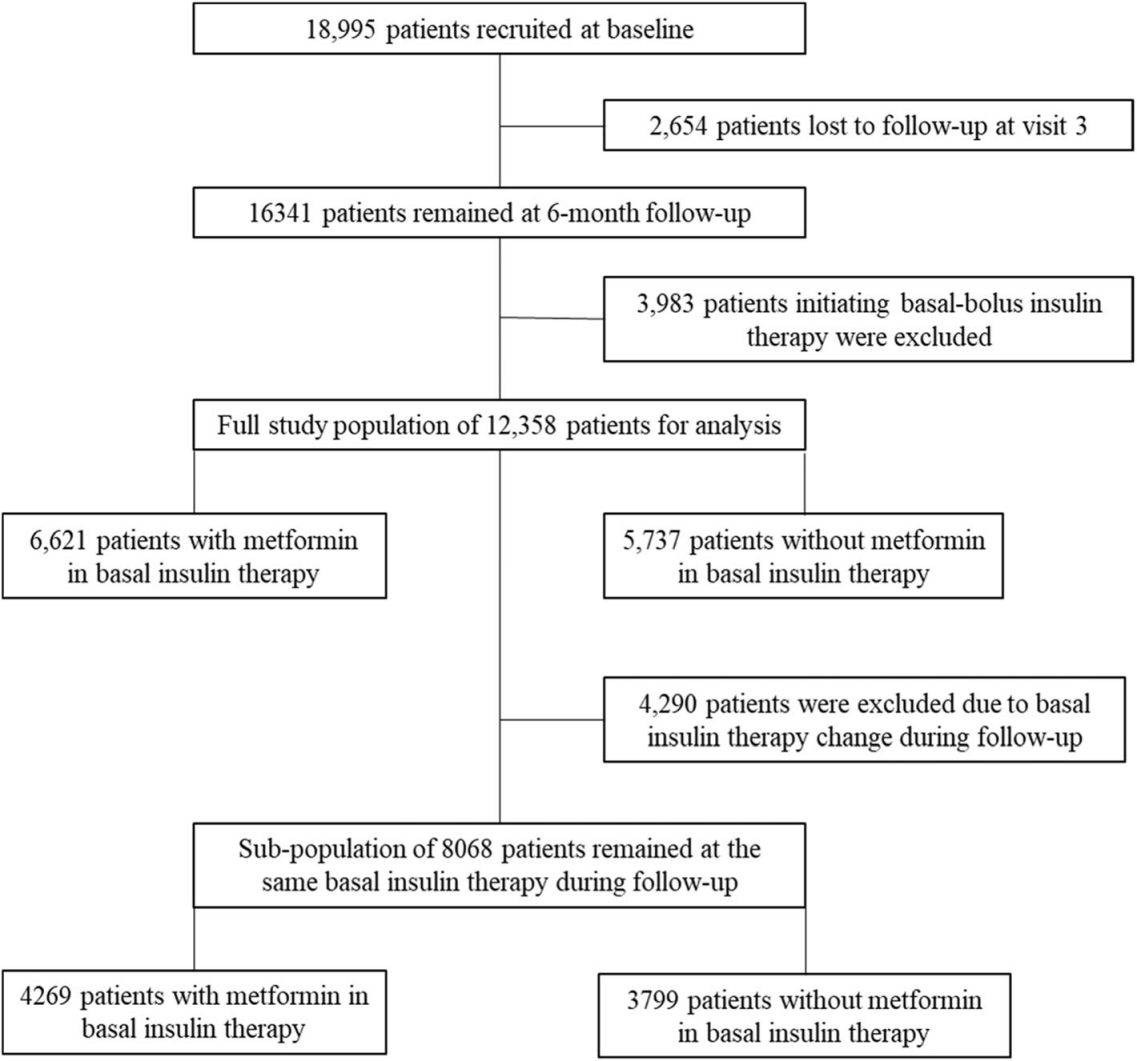
Flow of patients included in the analysis

Among all the recruited 18,995 participants at baseline, 12,358 (65.1 %) patients who initiated BI only therapy and completed 6 months follow-up were included in the analysis. Figure 1 shows the patients selection procedure.

### Patients’ characteristics and metformin use

Overall of the 12,358 patients, males accounted for 53.1 % and the mean age was 55.5 years. Patients had a mean diabetes duration of 6.2 years and OAD treatment duration of 5.5 years, BMI of 24.8 kg/m^2^, HbA1c of 9.3 %, and FPG of 11.2 mmol/L.

Among the full population, metformin was used by 65.2 % (8056/12,358), 53.6 % (6621/12,358) and 53.5 % (6611/12,358) of patients before, at the moment and after 6 months of BI initiation, respectively. When BI therapy was initiated, metformin was discontinued among 27.8 % (2243/8056) patients who had been using metformin previously. After 6 months follow-up, there were 14.1 % (934/6621) who were prescribed with metformin at baseline but stopped metformin in the end.

Before propensity score adjustments, baseline variables including age, recruitment settings (inpatient ward or outpatient clinic), current residence (urban or rural area), out of pocket ratio, BMI, diabetes and OAD treatment duration before BI therapy, systolic and diastolic blood pressure, HbA1c level at baseline, episodes of hypoglycemia, microvascular and macrovascular complications, number and types of OADs (metformin, α-glycosidase inhibitors and glinides) before BI therapy, and types of basal insulin at BI initiation were significantly different between BI with metformin and BI without metformin two groups. Balance of baseline characteristics between the two groups was achieved among most variables after propensity score adjustments except for metformin before BI therapy and episodes of hypoglycemia. (Table 1)

**Table 1:** Baseline characteristics of patients with basal insulin therapy before and after propensity score adjustment

	Before propensity score adjustment		After propensity score adjustment	
	BI with metformin (*N *= 6621)	BI without metformin (*N *= 5737)	BI with metformin (*N *= 6621)	BI without metformin (*N *= 5737)
**Demographics**				
Male (%)	52.8	53.4	53.1	53.1
Age (years, Mean ± SD)	54.2 ± 9.9**	56.8 ± 10.4	55.5 ± 10.9	55.4 ± 11.0
Education (%)				
Primary school or illiterate	24.9	26.0	25.2	25.7
Junior high school	30.8	29.2	30.3	29.8
Senior high school	25.6	26.2	25.9	25.9
Junior college	10.8	11.5	11.2	11.1
Bachelor’s degree or higher	7.9	7.1	7.4	7.5
Hospital level (%)				
Secondary hospitals	49.8	50.4	50.1	50.1
Tertiary hospitals	50.2	49.6	49.9	49.9
Recruitment settings (%)				
Outpatient clinic	54.5*	51.2	53.0	53.0
Inpatient ward	45.5	48.8	47.0	47.0
Current residence (%)				
Urban	69.1**	73.2	71.1	71.1
Rural	30.9	26.8	28.9	28.9
Out of pocket (%, Mean ± SD)	42.5 ± 28.1*	40.8 ± 27.2	41.7 ± 30.3	41.7 ± 30.7
**Clinical**				
BMI (kg/m^2^, Mean ± SD)	25.4 ± 3.3**	24.2 ± 3.2	24.9 ± 3.5	24.8 ± 3.5
Duration of diabetes (years, Mean ± SD)	6.2 ± 5.0*	6.5 ± 5.3	6.4 ± 5.6	6.4 ± 5.7
Duration of OAD treatment (years, Mean ± SD)	5.5 ± 4.9*	5.7 ± 5.0	5.6 ± 5.4	5.6 ± 5.5
SBP (mmHg, Mean ± SD)	130.1 ± 15.4*	129.3 ± 15.6	129.9 ± 17.0	129.4 ± 17.2
DBP (mmHg, Mean ± SD)	79.9 ± 9.2**	79.1 ± 9.2	79.4 ± 10.0	79.6 ± 10.2
HbA1c at baseline (%, Mean ± SD)	9.4 ± 1.8**	9.2 ± 1.8	9.3 ± 2.0	9.3 ± 2.0
FPG at baseline (mmol/L, Mean ± SD) (missing=616)	11.4 ± 3.5	11.0 ± 3.6	11.2 ± 3.8	11.2 ± 3.9
Hypoglycemia at baseline				
Percentage (%)	5.7	5.8	5.7	5.8
Episodes (times/100 person-year, Mean ± SD)	1.7 ± 9.6*	1.6 ± 9.9	1.7 ± 1.4*	1.6 ± 1.4
Complication at baseline (%)				
Macrovascular	13.5*	15.6	14.4	14.4
Microvascular	24.6*	27.5	25.9	25.9
Pre-insulin OAD type ^a^ (%)				
Metformin	87.8**	39.1	96.0*	97.3
Sulfonylureas	46.5	48.5	47.4	47.4
α-glycosidase inhibitors	18.1**	33.6	23.0	22.8
Glinides	13.4**	18.9	15.4	15.3
Thiazolidinediones	5.7	6.4	6.0	6.0
Others ^b^	8.7*	9.8	9.2	9.2
Pre-insulin OAD number at baseline (%)				
1	35.2**	51.9	43.0	43.0
2	50.9	40.9	48.1	48.2
≥3	13.9	7.1	8.9	8.8
BI type initiated at v1				
Glargine	69.6**	78.1	74.9	74.9
Detemir	14.4	12.5	13.7	13.7
NPH	16.0**	9.4	11.4	11.4
BI dose at v1 (U/day/kg)	0.17 ± 0.071	0.18 ± 0.069	0.18 ± 0.46	0.18 ± 0.47

*Abbreviation*: *BI* basal insulin, *BMI* body mass index, *OAD* oral antidiabetic drug, *SBP* systolic blood pressure, *DBP* diastolic blood pressure, *HbA1c* glycated hemoglobin, *FPG* fasting plasma glucose, *NPH* Neutral Protamine Hagedorn

**: <0.0001; *: <0.05

^a^Cumulative proportions might be over 100 % as some patients might use two or more OADs

^b^Others included DPP-IV inhibitors, Aldose reductase inhibitors, GLP-1 receptor agonists, Compound drugs, and Traditional Chinese medicine

### Confounding factors after 6-month follow-up

Table 2 listed the comparison between patients initiating basal insulin with and without metformin for both full population analysis and sub-analysis, including number of OADs, insulin dose, diet and physical exercise status after 6-month follow-up at visit 3. In full and sub-populations, all the listed factors at visit 3 were significantly different between two groups.

**Table 2:** Comparison of diabetes management between patients initiating basal insulin with and without metformin after 6-month follow-up at visit 3, (Mean ± SD)

	BI with metformin	BI without metformin
**Full population initiating BI therapy (*N*= 12,358)**	***N*= 6621 (53.6 %)**	***N*= 5737 (46.4 %)**
OAD number per day at visit 3	1.63 ± 0.76	1.19 ± 0.74
Metformin (%)	85.9 %	16.1 %
Sulfonylureas (%)	30.3 %	27.8 %
Glinides (%)	18.3 %	28.6 %
α-glycosidase inhibitors (%)	23.5 %	45.3 %
BI dose at visit3 (U/day/kg)	0.16 ± 0.12	0.18 ± 0.11
Insulin dose at visit3 (U/kg/day)	0.18 ± 0.13	0.20 ± 0.13
Staple food at visit3 (g)	161.16 ± 44.02	156.15 ± 42.69
Vegetables at visit3 (g)	188.08 ± 66.49	184.61 ± 64.72
Fruit at visit3 (g)	100.17 ± 42.76	98.62 ± 39.86
Meat at visit3 (g)	104.94 ± 37.26	103.09 ± 35.21
Days of participating in >30 min physical activity in past 7 days at visit3	5.98 ± 1.99	5.86 ± 2.09
Days of participating in specific exercise session in past 7 days at visit3	4.11 ± 2.91	4.00 ± 2.90
**Sub-population keeping BI therapy during 6-month follow-up (*N *= 8068)**	***N*= 4269 (52.9 %)**	***N*= 3799 (47.1 %)**
OAD number per day at visit 3	1.75 ± 0.65	1.08 ± 0.66
Sulfonylureas (%)	32.1 %	28.1 %
Glinides (%)	17.8 %	28.6 %
α-glycosidase inhibitors (%)	21.6 %	46.1 %
BI dose at visit 3 (U/day/kg)	0.21 ± 0.09	0.21 ± 0.09
Staple food at visit 3 (g)	161.37 ± 43.61	154.81 ± 41.17
Vegetables at visit 3 (g)	185.73 ± 65.28	180.62 ± 63.87
Fruit at visit 3 (g)	101.23 ± 41.63	98.59 ± 38.42
Meat at visit 3 (g)	104.63 ± 36.96	101.54 ± 34.38
Days of participating in >30 min physical activity in past 7 days at visit 3	5.98 ± 1.97	5.83 ± 2.10
Days of participating in specific exercise session in past 7 days at visit 3	4.17 ± 2.88	3.99 ± 2.87

All the listed factors at visit 3 were significantly different between two groups in full and sub-populations

*Abbreviations*: *BI* basal insulin, *NPH* Neutral Protamine Hagedorn, *OAD* oral antidiabetic drug

#### Primary outcomes

In the full population analysis, control rates of HbA1c≤6.5 % and HbA1c <7.0 % were both significantly higher in BI with metformin group (25.0 % and 39.9 %) than those in BI without metformin group (22.1 % and 36.4 %) (*P *= 0.0024; *P *= 0.0011) (Table 3). No significant differences were shown in terms of hypoglycemia between two groups at visit 3. The statistical significance of primary outcomes between two groups in the sub-analysis was consistent with that in the full population analysis, with higher control rates of HbA1c≤6.5 % (30.1 % vs. 25.1 %, *P *= 0.0003) and HbA1c <7.0 % (47.9 % vs. 41.9 %, *P *= 0.0001) in BI with metformin group (Table 3).

**Table 3:** Effectiveness and safety of BI with or without metformin after 6 months - results of propensity score regression^a^

Dependent variables	BI with metformin	BI without metformin	*P* value
**The full population (*N *= 12,358)**	***N *= 6621**	***N *= 5737**	
HbA1c control, % (95 % CI)			
≤6.5 %	25.0 (20.9, 29.5)	22.1(18.4, 26.3)	0.0024
<7.0 %	39.9 (34.1, 45.0)	36.4 (31.7, 41.3)	0.0011
HbA1c level (%), mean (95 % CI)			
Mean level at v3	7.4 (7.3, 7.6)	7.5 (7.4, 7.6)	0.0302
Change from baseline	-1.8 (-1.9, -1.6)	-1.7 (-1.9, -1.5)	0.1839
FPG control (<7.0 mmol), % (95 % CI)	36.0 (31.2, 41.0)	33.5 (28.9, 38.5)	0.0515
FPG level (mmol/L), mean (95 % CI)^b^			
Mean level at v3	8.3 (8.1, 8.5)	8.3 (8.1, 8.5)	0.6875
Change from baseline	-2.9 (-3.3, -2.5)	-3.0 (-3.3, -2.5)	0.5169
Insulin dose (U/day/kg), mean (95 % CI)			
Insulin dose at v3	0.33 (0.32, 0.33)	0.34 (0.33, 0.34)	<0.0001
Change from v1 to v3	0.064 (0.059, 0.060)	0.068 (0.063, 0.070)	0.0035
Weight (kg), mean (95 % CI)			
Mean level at v3	70.1 (69.3, 70.0)	70.3 (69.4, 71.2)	0.4862
Change from baseline	0.35 (0.11, 0.59)	0.37 (0.13, 0.62)	0.7223
Hypoglycemia, % (95 % CI)	11.2 (9.1, 13.8)	10.4 (8.4, 12.8)	0.2965
**The sub-population (*N *= 8035)**	***N *= 4269**	***N *= 3799**	
HbA1c control, % (95 % CI)			
≤6.5 %	30.1 (28.4, 31.8)	25.1 (23.4, 26.8)	0.0003
<7.0 %	47.9 (46.1, 49.7)	41.9 (39.9, 43.8)	0.0001
HbA1c level (%), mean (95 % CI)			
Mean level at v3	7.2 (7.2, 7.3)	7.3 (7.3, 7.4)	0.0009
Change from baseline	-2.0 (-2.1, -1.9)	-1.9 (-2.0, -1.9)	0.4175
FPG control (<7.0 mmol), % (95 % CI)	49.6 (47.5, 51.8)	46.8 (44.5, 49.1)	0.1319
FPG level (mmol/L), mean (95 % CI) ^c^			
Mean level at v3	7.5 (7.4, 7.6)	7.6 (7.5, 7.7)	0.2212
Change from baseline	-3.7 (-3.8, -3.5)	-3.7 (-3.9, -3.5)	0.8499
Insulin dose (U/day/kg), mean (95 % CI)			
Insulin dose at v3	0.21 (0.20, 0.21)	0.22 (0.21, 0.21)	<0.0001
Change from v1 to v3	0.033 (0.031, 0.035)	0.037 (0.035, 0.039)	0.0073
Weight (kg), mean (95 % CI)			
Mean level at v3	67.4 (67.0, 67.8)	67.7 (67.3, 68.1)	0.2965
Change from baseline	-0.12 (-0.22, -0.02)	0.15 (0.042, 0.24)	0.0013
Hypoglycemia, % (95 % CI)	7.2 (6.3, 8.2)	6.9 (6.0, 8.0)	0.6892

*Abbreviations*: *v1* baseline, visit 1, *v3* 6-month, visit 3

^a^Independent variables include propensity score, process confounding factors and metformin group. Baseline covariates balanced by propensity score adjustments between two groups include regions, hospital level, gender, age, current residence, medical insurance (actual proportion of out of pocket), BMI, diabetes duration, OAD treatment duration, HbA1c level and FPG level at baseline, diabetes complication, number and types of OADs before BI therapy, BI types at BI therapy initiation. Process confounding factors controlled in the models include the number of OADs and total insulin dose (uncontrolled in the model when analyzed as the outcome) at visit 3, physical activity and diet consumption

^b^4018 patients had missing data for FPG level with 2190 patients from BI with metformin group and 1828 patients from BI without metformin group

^c^2440 patients had missing data for FPG level with 1306 patients from BI with metformin group and 1134 patients from BI without metformin group

### Secondary outcomes

In the full population analysis, BI with metformin group had lower adjusted mean of insulin dose at v3 (0.33 vs. 0.34 U/day/kg, *P *< 0.0001), lower increment of insulin dose from v1 to v3 (0.064 vs. 0.0068 U/day/kg, *P *= 0.0035), and lower HbA1c mean level at v3 (7.4 % vs. 7.5 %, *P *= 0.0302) compared with BI without metformin group. Weight, HbA1c and FPG levels changes from v1 to v3 and adjusted mean levels at v3 of FPG and weight were not significantly different between the two groups, as well as FPG control rate (<7.0 mmol/L). (Table 3)

In the sub-analysis for patients remained at the same BI therapy consistently with or without metformin during 6-month follow-up, BI with metformin group also had a lower insulin dose at v3 (0.21 vs. 0.22 U/day/kg, *P *< 0.0001), less increment of insulin dose from v1 to v3 (0.0033 vs. 0.0037 U/day/kg, *P *= 0.0073), and lower level of HbA1c (7.3 % vs. 7.2 % *P *= 0.0009) compared with BI without metformin group. The sub-analysis showed BI with metformin group had slightly weight reduction while BI without metformin group had weight gain at v3 (-0.12 vs. 0.15 *P *= 0.0013). No significant differences were observed in mean levels of FPG and weight, FPG control rate (<7.0 mmol/L), as well as HbA1c and FPG levels changes from v1 to v3 in the sub-analysis. (Table 3).

## Discussion

This real-world study observed that around half of T2DM patients initiating BI therapy due to uncontrolled hyperglycemia on OADs were prescribed concomitantly with metformin. BI therapy with metformin showed higher rates of glycemic control, less increment of insulin dose and less weight gain compared with BI without metformin after 6-month follow-up. These results suggest that metformin-based BI therapy regimen can be advantageous for patients with suboptimal glycemic control on OADs in improving glycemic control and reducing weight gain.

In this real-world study, the overall proportion of patients using metformin prior to BI therapy was 65.2 %, in which 72.2 % continued metformin after BI therapy. After BI therapy, the overall proportion of metformin use was 53.6 %. An observational study done in the US reported more than 70 % patients taking metformin before insulin therapy and 80.3 % patients continued it after insulin initiation. The proportion of metformin use after insulin therapy was also higher (63.8 %) in Pilla et al.’s study [19]. Another study with commercial insurance data also reported 72.7 % patients taking metformin before insulin initiation and metformin was continued in 84.6 % patients after insulin therapy [20]. Given its low price, safety profile, and potential cardiovascular protection, metformin is recommended as first-line glucose lowering drug for treating T2DM unless contraindication or intolerance, which is also suggested being continued in combination with other agents including insulin [9, 10, 25]. Nevertheless, 35.8 % of patients were not treated by metformin before BI therapy and 27.8 % patients stopped metformin after initiating BI in our study, which may suggest the underuse of metformin in Chinese patients with T2DM in spite of possible contraindication or intolerance among some patients.

We did not directly collect the reasons from the participants and physicians for metformin discontinuity after BI initiation, including data for renal or hepatic contraindications to metformin, due to the study was mainly focus on insulin therapy by design. However, our current data can provide some clues for further discussion and research. (1) Renal contraindication to metformin should be among the reasons but seems not the most important one. There were only 11.6 % (259/2242) of metformin-discontinuers were confirmed with diabetic nephropathy at baseline and most of them should be at the stage of mild-to-moderate renal impairment. Meanwhile, 7.3 % of metformin-continuers were detected with diabetic nephropathy. (2) It seems common for physicians to replace metformin by other medications, most likely to address the postprandial glucose. Among those metformin-discontinuers, most (76.4 %) were prescribed with α-glycosidase inhibitors, glinides and short acting sulfonylureas. This seems to be a routine practice with no significant difference between in-patient and out-patient settings (52.7 % vs. 47.3 %). (3) Some clinicians tend to stop any oral agents for patients with relatively less severe diabetes. Among metformin-discontinuers, 23.6 % patients who stopped any OADs at v3 had lower HbA1c level (9.07±1.82 % vs. 9.36±1.88 %, *p *= 0.0014) and took fewer OADs (40.9 % vs. 29.5 % with only one OAD before BI initiation, *p *< 0.0001) at baseline when compared to those with any oral agents at v3.

The reimbursement policy of insulin therapy (human insulin versus insulin analogues) in China should not affect the results. All the insulins, human insulin and insulin analogues, reported in ORBIT study have been covered in the reimbursement policy in China. The reimbursement ratio differs from inpatient or outpatient settings, urban or rural, with NCD card or nor, etc. However, all these key impact factors as well as the exact ‘out of pocket’ ratio have been well balanced between the two study groups (BI with metformin group and BI without metformin group) after propensity score adjustment (Table 1). Further studies about factors associated with metformin discontinuation after BI therapy are required.

In our study, the sub-analysis tends to provide higher estimates of the effectiveness and safety for BI with metformin over without metformin than the full population analysis. Patients in full population analysis who switched to other treatment regimens might be those with suboptimal glycemic control at the initial BI treatment. Therefore, compared with sub-analysis, the full population analysis containing patients that switched treatment regimen during follow-up may also dilute inter-group differences if a higher proportion of patients switching to other regimens in BI with metformin group remained having suboptimal glycemic control than those in BI without metformin group. In terms of safety, as patients could not tolerate prescribed regimen or experienced adverse events, they tend to not continue with the initial regimen. In this way, exclusion of these patients in the sub-analysis might make a study drug appear safer than it really is. However, given the wide acknowledged safety profile of metformin [12], risks of hypoglycemia or other adverse effects caused by BI with metformin should be low in our study. Despite of some discordances between the full population analysis and the sub-analysis, they both illustrated the advantages of metformin-based BI therapy in increasing glycemic control and insulin dose.

Most previous randomized studies reported the superiority of insulin combined with metformin in reducing HbA1c, weight gain, insulin dose or hypoglycemia compared with insulin alone or insulin plus other OADs, whereas they were not always concordant with each other [14, 16–18]. In a study by Hollander et al., insulin plus metformin was reported to be associated with more HbA1c reduction, less weight gain and lower hypoglycemia rates compared with insulin plus sulfonylurea [17]. Nevertheless, in a study by Lund et al., insulin plus metformin showed less weight gain (2.22 vs. 4.73 kg) but equivalent HbA1c reduction (from 8.15 ± 1.32 to 6.72 ± 0.66 % vs. 8.07 ± 1.49 to 6.90 ± 0.66 %) compared with insulin plus repaglinide [26]. Strowig et al. also reported lower weight gain (0.5 vs. 4.4 kg) and fewer hypoglycemic episodes (0.6 vs. 2.0/1.7 episodes/person/month) in insulin plus metformin group compared with insulin alone or insulin plus troglitazone but a comparable average reduction in HbA1c with insulin plus metformin as with insulin alone (both from 8.8 ± 1.2 to 7.1 ± 1.0 %) [16]. However, previous randomized studies did not specify the type of insulin as basal insulin [16, 26] or the participants as insulin-naïve patients who initiated BI therapy due to suboptimal glucose control on OADs [17]. Given the current guidelines consistently recommend timely initiating insulin therapy when glycemic target cannot be maintained by OADs in addition to lifestyle management, and that BI is considered as the most appropriate initial insulin regimen, [9, 10] this real-world study correspondingly investigated the effectiveness and safety of initiating BI plus metformin in Chinese T2DM patients of which the results can further support its application in China. However, it is worth noting that the clinical effect size of initiating BI plus metformin found in this real-world study was small. The HbA1c difference between the two groups was 0.1 %, and the insulin dose achieved was only 0.01u/kg/day. These indicate that even prioritizing metformin, many other aspects regarding diabetes management should be also considered, such as improved blood glucose monitoring, non-drug treatment, therapy adjustment and dose titration.

Strengths of this analysis are its large size, wide geographic variation and reliance on data from prospective, real-world clinical practice, which make the findings in our study more representative for a broader population of those patients and more capable of reflecting actual medication practice and its effectiveness and safety than in traditional randomized clinical trials. In addition, appropriate adjustments for various potential clinical and non-clinical factors improved the robustness of this statistical analysis.

One major limitation of this study was the possibility of confounding, which was one of the common flaws in observational studies. Although propensity score-adjusted regression model was applied to address confounding by covariates of baseline characteristics and factors at 6-month follow-up were also controlled, some differences between groups may be still present. Moreover, given its large size, the statistical difference may not necessarily conclude the clinical relevance. Another limitation is that data on eGFR reflecting renal function, liver function and gastrointestinal side effects were not collected. Finally, as this study only evaluated the outcomes of glycemic control, weight and hypoglycemia due to the short follow-up period, the impact on long-term outcomes such as microvascular or macrovascular complications and death were not assessed. Since this study was conducted in 2011 to 2013, which was in the pre-SGLT2 inhibitor and GLP1A era in China, the use of the two agents were rarely reported. Considering that the use of SGLT2 inhibitors may delay the need for insulin initiation, and the use of GLP1A is now prioritized over basal insulin initiation, the BI initiation might be very different now.

## Conclusions

In conclusion, adopting basal insulin therapy with metformin in T2DM patients with previously suboptimal glycemia control by OADs was associated with better glycemic control and probably lower insulin dose and less weight gain compared with BI without metformin in the real-world setting, although the clinical relevance of these results must be interpreted with caution. Considering the low use rate prior to BI initiation and high discontinuing rate after BI initiation of metformin, in-depth analyses should be conducted to explore the reasons and intervention strategies should be developed and evaluated to promote metformin use in China.

## Data Availability

The data used to support the findings of this study are available from the corresponding author upon request.

## Abbreviations

BI: Basal insulin
BMI: Body mass index
CI: Confidence intervals
DBP: Diastolic blood pressure
FPG: Fasting plasma glucose
HbA1c: Glycated hemoglobin
IRB: Institutional Review Board
NPH: Neutral Protamine Hagedorn
ORBIT: Observational Registry for Basal Insulin Treatment
OAD: Oral antidiabetic drug
T2DM: Type 2 diabetes mellitus
SBP: Systolic blood pressure
SD: Standard deviation
